# The relationship between knowledge of ergonomic science and the occupational health among nursing staff affiliated to Golestan University of Medical Sciences

**Published:** 2010

**Authors:** Leila Juibari, Akram Sanagu, Nafiseh Farrokhi

**Affiliations:** * Assistant Professor, School of Nursing and Midwifery, Golestan University of Medical Sciences, Gorgan, Iran; ** Nursing, School of Nursing and Midwifery, Golestan University of Medical Sciences, Gorgan, Iran

**Keywords:** Ergonomics, nurses, occupational accidents

## Abstract

**BACKGROUND:**

Occupational hazards are much higher for nurses than many other jobs and neglecting this fact may reduce the quality of nursing services. The aim of this study was to investigate the relationship between knowledge of ergonomics and occupational health among the nursing staff affiliated to Golestan University of Medical Sciences.

**METHODS:**

It was a cross-sectional analytical study on 423 nursing staff working in various medical centers affiliated to Golestan University of Medical Sciences in 2008, selected by quota randomized sampling. Data collection instrument was Ergonomics Questionnaire, which consisted of 72 questions. Cronbach’s alpha for main sections of the questionnaire was 0.8, 0.8 and 0.9. Descriptive and analytical tests were used for data analysis and an alpha error of 5% was considered.

**RESULTS:**

Of all the subjects, 36.1% had 5-10 years of work experience, 61.9% had a good knowledge of ergonomic principles, and 83% were exposed to a mild level of occupational hazards. There was no significant relationship between knowledge of ergonomics and occupational health (p = 0.08). The relationships between knowledge of ergonomics and age, gender, marital status, work experience, the type, and the location of service were significant (p < 0.05). The relationship between occupational health and age, work experience, employment type, and location of service were also statistically significant (p < 0.05).

**CONCLUSIONS:**

Training staff to do their job in the best way, taking breaks between long working hours, using appropriate tools and facilities and paying attention to ergonomics can provide a healthier work environment for nurses and optimize human resource efficiency.

In the past century, the development of science and technology have led to the economic growth in most industrial countries. Most evidences especially in the developing countries showed that the lack of balance between the technology and its users in an environment where technology is used causes negative outcomes such as less and lower quality of products and higher injuries and job incidence rates.^1^ Most of these problems in job environments can be solved using ergonomic methods. Ergonomics is the science of relationship between human, environment and machine and technology, trying to improve their relationship and making a balance between them. Ergonomics assess and evaluate human abilities and help engineers and designers to build systems and processes in balance with human characteristics.^2^,^3^ Some evidences say that application of ergonomics in designing mechanical process and systems have had significant effects on increasing products, decreasing medical expenses, reducing psychological pressure, increasing job satisfaction, increasing efficiency and in general, and increasing national incomes and economic benefits. Improving health care services and hospital processes is not possible without cooperation of all human resources and smoothing, continuous, favorable and effective services. Among human resources working in hospitals, nursing activities are more important than others because they provide medical care to patients and have more relationship with them compared to other groups. Providing high quality of nursing care is related to the number and quality of nurses who work in a hospital. Nursing career has a nature of 24 hours dealing with patients therefore, it is hard, exhausting and is associated with stress and psychological pressure. Occupational hazards for nurses are very high and mainly are related to the pressures of the job and non-standard work facilities.^4^ Neglecting nursing staff may reduce the quality of nursing services. Therefore, following standard conditions of working environment can decrease the job pressure for nurses. Implementing ergonomics in the world has faced many challenges, which are not only in the developing countries like Iran. Due to lack of familiarity with the advantages of using this science in organizations, there is a resistance towards ergonomics changes.^5^

The aim of this study was to determine the relationship between knowledge of ergonomics sciences and occupational health of the nursing staff working in Golestan province.

## Methods

This was a cross-sectional analytical study to assess the working condition of nursing staff, determining their working problems and occupational hazards and their knowledge of errollcs applied science in 2008 in nursing units of Golestan province and in the hospitals affiliated to the Golestan University of Medical Sciences. The study population included all nursing staff of the hospitals including head-nurses (matrons), nurses, assistant nurses and health care workers. All the nursing staff with working experience of less than a year, and birth defects, disabilities or injuries from out of workplace was excluded. Also, all health related off days unrelated to hospital works such as delivery off time were not considered.

Considering the standard deviation of 0.7 and error level of 0.06 and with 95% certainty, a sample of 423 nursing staff were selected using random sampling, from both male and female staff who met the inclusion criteria and the questionnaire was sent to them.

The questionnaire consisted of 77 questions in 5 sections. The first section included demographic data, and the second section included 13 closed questions in Likert scale assessing knowledge of ergonomics; the third section included 16 closed questions and 5 open questions assessing the working conditions, the fourth section included 22 closed questions in Likert scale and 10 open questions assessing work injuries of nursing staff during the past year and the fifth section included 3 open questions.

Validity of the questionnaire was proved by consulted specialists and professors of nursing, health care and managers of health and medicine and its reliability was proved in the pilot study. Cronbach’s alpha of the sections on the knowledge of ergonomics, working conditions, working problems and job injuries were 0.8568, 0.8223, and 0.9204 respectively.

Questions to assess knowledge of ergonomics were related to the knowledge about correct body position when carrying objects, working with sharp equipments, correct transportation of patients, reducing tiredness of work and questions of working conditions were related to the light, air conditioning, temperature conroll, quiet working environment; questions of working problems and job injuries were related to having muscle-skeleton complications, infective diseases, depression, sleeplessness, working stress and etc.

The answers to the questions in section 2, 3 and 4 were scored from 0 to 5. Questions on knowledge of ergonomics, working conditions and job injuries were categorized from very weak to very good. This questionnaire was previously used in another study in Iran.^4^

Data were analyzed using SPSS. For quantitative data mean and standard deviation were used and for qualitative data absolute and relative frequency were used. To determine the significance of relationship between variables, chi-square, independent t, Pearson and Spearman tests with error level of 5% were used.

Ethical consideration were followed by taking permission from research deputy, hospital authorities, and taking written consent from participants anonymously on the questionnaires, secrecy of the information and giving the right to leave the study at any time.

## Results

Out of 423 nursing staff of 7 hospitals in Golestan province, who participated in the study, 71.1% were women, 33.7% were 25 to 30 years old, 94.9% had an undergraduate degree, and 36.1% (149) had 5 to 10 years of working experience.

Findings showed that in the past year, 83% (341) were exposed to mild working problems and job injuries and 4.6% were exposed to severe injuries; 82.8% (342) of nursing staff described the working conditions in educational hospitals average.

Knowledge of ergonomics in 255 nurses was high. As the nurses’ knowledge of ergonomic science increased, the rate of working injuries among them obviously decreased. However, there was no statistically significant relationship between these two variables (p = 0.08).

In demographic variables, the relationship between knowledge of ergonomics and age (p = 0.004) and working experience were significant (p < 0.001). As the working experience of nurses increased, their knowledge was increased as well. The relationship between knowledge of ergonomics and employment rank and location was also significant; (p = 0.006 and 0.02 respectively). There was no significant relationship between knowledge of ergonomics and sex, marital status, educational degree and working location (p > 0.05).

Data analysis showed a significant relationship between job injuries of nurses and their age (p = 0.00); as they were older, they had more job injuries. Also, there was a significant relationship between job injuries and work experience (p = 0.00), employment rank (p = 0.00) and the hospital they were working in (p = 0.029). However, there was no significant relationship between job injuries of nurses and their gender (p = 0.4), marital status (p = 0.7), educational degree (p = 0.2) and working problems (p = 0.06).

Emergency rooms, internal medicine, infectious and infant’s wards were respectively the most wards where the nurses were working during the past year. There was no significant relationship between occupational health and service sector location (p = 0.64), although most injuries was related to working in the emergency rooms and ICU. Of all, 20.3% were standing on their feet for at least 5 hours per working shifts, while 50 percent had a maximum of one-hour sitting time during each shift; 22.5% of nurses reported three consecutive shifts of working; and 38.3% said that they were injured 5 to 10 times more with sharp instruments such as needles, razors, and scissors in the past year.

Most complications were related to the leg and back (46.9%) and the least were related to the fingers (0.5%). About 50% of the nurses reported that their musculoskeletal problems are caused through their works while standing on their feet and 16% said that they were related to insufficient education.

Nearly 60% (246) of nurses described that the most notable musculoskeletal symptom is pain; 10.9% of nurses during the past year had at least two days off due to medical complications, but 51.8% of nurses never used their off days due to shortages of human resources.

## Discussion

In the present study, no significant relationship was found between the knowledge of ergonomic principles and occupational health of nurses. This finding may be interpreted as follows: when a working environment is not established based on ergonomic principles, the knowledge of staff cannot be the only success factor.^6^ Infectious diseases can be one of the occupational injuries. Half of the nurses said that they suffered from infectious diseases due to their job. Also, a significant percentage of subjects suffered from physical injuries with sharp instruments such as needle, razor, etc. Unfortunately this rate was high in similar studies in Iran.^4^ More than half of the subjects suffered from physical injuries when transporting patients, which was similar to the results of the studies in other countries. Considering the young age of nursing personnel in Golestan province, physical injuries can cause early fatigue of nurses, which is a loss of national resources. Worn out equipments and supplies at the university hospitals can increase the problem too. According to some sources, nurses and health care personnel experience annually more than 200 thousand cases of occupational injuries and the highest risk of this occupation is physical and ergonomics factors due to moving and transporting patients.^7^

More than half of the studied nurses were attacked and threatened by patients and their relatives, which is unfortunately also confirmed by other studies. Health care environments should be safe for service providers in addition to patients; they must also have the necessary safety. But the findings confirmed the opposite. On the other hand, about sixty percent of the nurses were depressed in the past year, due to their work and in a similar study, this rate has been close to 90 percent.^4^ Insomnia and sleep disorders (nightmares) related to work was also significant in the present study. In Tirgar’s study also out of 197 samples, 84 percent complained about the sleep disorders.^8^

Even though there are not many studies on occupational injuries of nurses, similarities in results of studies in different parts of Iran can be a sign of an epidemic problem in all Iranian medical centers, which requires special attention by authorities to make serious reforms in health care centers in Iran.

Ergonomic problems in nursing have turned to a global phenomenon. A study of 125 nurses in Taiwan showed that within one year, about 36.8% of nurses suffered from musculoskeletal problems.^9^–^12^ Transferring patients from bed to stretcher, turning the patient around on the bed, changing patients’ clothes and bed sheet were important causes of musculoskeletal injuries in nurses.^13^,^14^ Thus, it is expected that by optimizing equipments and simplifying the caring labors the incidence of occupational injuries might be reduced. Although these findings do not seem new, they are important and despite being a heavy load on the health system, they have been neglected.

Using intervention techniques can be effective in reducing ergonomic related problems. It was shown in a study that using an intervention program has been effective on stress, trauma and patient care. Five years after the intervention program, back and shoulder injuries and absence from work and the need for leave of absence were still declining, while there was no improvement in the control group.^15^,^16^ The findings of this study showed that the majority of nursing personnel, have had experienced working for more than one shift continuously. In a longitudinal study on 10793 work force, it was determined that working in consecutive hours is associated with 61 percent risk of occupational hazards^17^ and a study on a group of dentists also showed that 49.5% of these physicians did not considered a break time for themselves between visiting patients, while the relationship between taking breaks and the incidence of musculoskeletal pain was significant.^18^

## Conclusion

Experts believe that the existing challenges are due to the lack of humanistic thinking in designing work systems, because ergonomics is not yet a requirement in organizational life. At the first step, the industrial society should be oriented towards implementation of ergonomics. Then, the need for ergonomics will develop in the organization; and after that the professional level of preparing implementation methods and ergonomics standards and localizing them based on the needs of organizations can be started.^5^

The results of this study confirmed that a significant number of nursing personnel had been suffering from a mild level of occupational injuries and musculoskeletal symptoms. The present findings and existing literature showed that a staff who trained to do their job properly and in the best way, the importance of having breaks between long hours of working, using appropriate equipments and facilities and authorities’ who pay more attention to the ergonomic considerations, particularly in emergency rooms and intensive care units (ICU), can provide a healthy work environment.

The authors declare no conflict of interest in this study.
